# A Phenomenological Study of Postgraduate Medical Trainees’ Incidental Learning Experiences and Psychological Well-Being During the COVID-19 Pandemic

**DOI:** 10.7759/cureus.54937

**Published:** 2024-02-26

**Authors:** Vernon R Curran, Ann Hollett, Karla Simmons, Teri Stuckless, Greg Radu

**Affiliations:** 1 Faculty of Medicine, Memorial University of Newfoundland, St. John's, CAN; 2 Radiation Oncology, Memorial University of Newfoundland, St. John's, CAN

**Keywords:** qualitative, interviews, graduate medical education, learning, psychological well-being, covid-19

## Abstract

Background: During the COVID-19 pandemic, postgraduate medical trainees contributed significantly to the healthcare workforce, as multiple vulnerabilities in the healthcare system and medical training were expounded. The burden of training, learning, and working at this time introduced unique psychological and emotional stressors within a context of generalized volatility and radically different ways to work and learn. This study explored postgraduate trainees’ experiences with coping, managing, and learning during the COVID-19 pandemic.

Methodology: Using a phenomenological approach, semistructured interviews were conducted with an intradisciplinary sample (*n *= 8) of postgraduate trainees in Newfoundland and Labrador, Canada, between May and October 2022. Five researchers performed inductive and deductive thematic analysis to develop a coding structure and identify common themes.

Results: The COVID-19 pandemic prompted the use of restrictive public health measures and an unprecedented shift from in-person to virtual learning. This affected trainees’ exposure to normalized learning experiences, training rotations, and opportunities to learn from peers and staff. Certainly, trainees reported that virtual learning improved their educational experiences in unique ways, increased engagement and attendance, and enabled regular meetings and learning when in-person options were unavailable. Trainees also reported enhanced self-directed learning skills, greater ownership of and leadership in their education, and increased confidence and experience with virtual care. Some also reported a perceived increase in elements of emotional intelligence (e.g., self-awareness, empathy, and compassion).

Conclusions: Trainees reported a variety of incidental learning experiences from working and training during COVID-19. Although some experiences were challenging, there was a perception that such experiences led to new learnings that were beneficial to one’s professional development and future career, as well as implications for future training provided to trainees. While there was a reported shift in the culture surrounding postgraduate trainees' health and safety, respondents also described the need for additional support for postgraduate trainees' well-being during a pandemic.

## Introduction

Beginning in early 2020, COVID-19, a novel pneumonia caused by SARS-CoV-2, began to spread across the globe. Since then, COVID-19 has had a considerable impact on healthcare systems worldwide and has threatened the physical, psychological, and social health of healthcare workers (HCWs) [1-3]. HCWs were particularly vulnerable to emotional distress during COVID-19 and frontline staff directly involved in the diagnosis, treatment, and care of patients were at risk of developing psychological distress and other mental health symptoms. Kisely et al.’s [4] rapid review and meta-analysis found that HCWs in contact with COVID-19-affected patients had greater levels of both acute or post-traumatic stress and psychological distress. Younger, more junior individuals, parents of dependent children, and those lacking practical support were associated with greater risk factors for psychological distress [4]. The increasing number of confirmed and suspected cases, overwhelming workload, depletion of personal protection equipment (PPE), lack of specific drugs, isolation, and *fear of possible infection in themselves and their families* may have all contributed to the mental burden of these HCWs [2,5-10].

Postgraduate trainees in Canada are medical doctors who have graduated from a Canadian or an international medical school and have entered the vocational aspect of their training pathway. These doctors play an important service role in the Canadian healthcare system in the frontline provision of clinical care. In usual times, they are among the most vulnerable, with a high prevalence of burnout and psychological morbidity [2,11-12]. During COVID-19, postgraduate trainees were likely to contribute significantly to the workforce required to manage the increasing volume of patients with COVID-19. They were often redeployed, and rotations were modified, moved, or suspended [13-14]. Onboarding and orientation of new postgraduate trainees changed as well, and those moving from locations with a high prevalence of COVID-19 may have been required to temporarily self-isolate [15]. The transfer and reassignment of postgraduate trainees to high-demand departments, as well as the increased likelihood of contracting suspected or confirmed cases of COVID-19, have been identified as some of the concerns expressed by postgraduate trainees during the COVID-19 pandemic [2].

Imran et al.’s [2] study of the psychological impact of COVID-19 found that the COVID-19 outbreak led to an increased prevalence of depressive symptoms and anxiety among postgraduate trainees. Female postgraduate trainees, senior trainees, and frontline workers reported experiencing anxiety, depression, and acute stress symptoms. Depression in postgraduate trainees was found to be associated with increased workload, sleep deprivation, and junior resident status. These stressors were compounded by a high risk of infection; inadequate safety equipment; social isolation, especially from family; and physical exhaustion [2,15-17]. Female postgraduate trainees reported a higher prevalence of psychological morbidity as well as more severe symptoms on all psychological measures, including depression, anxiety, and acute stress disorder [2].

While protecting HCWs became an important component of public health measures during COVID-19, there is a need for a better understanding of the nature and effect of the pandemic on postgraduate trainees' well-being and the nature of supports and interventions that may have been effective or could be supplemented to help postgraduate trainees with their psychological well-being. This research is important for our current and future postgraduate trainees. Imran et al. [2] recommend that further research is needed to assess the long-term impact of the COVID-19 outbreak on trainees' mental health as well as the effectiveness of any interventions to improve their psychological well-being. Qualitative research adds a unique dimension to understanding postgraduate trainees’ experiences during the current COVID-19 pandemic and how they have been affected and empowered to cope.

According to Prideaux [18], the curriculum exists at three levels in medical education: the planned curriculum for learners, the curriculum delivered to learners, and the curriculum as experienced by the learners. The planned curriculum includes what is intended by the designers, while the delivered curriculum involves what is organized by administrators and taught by medical teachers. The experienced curriculum encompasses what is learned. Eisner [19] suggests that what is planned and delivered may also be referred to as the explicit curriculum. However, what is learned may also encompass the unplanned or ‘hidden’ curriculum, which refers to the unwritten, unofficial, and often unintended values, views, and lessons that are learned in educational environments [20]. Some of the published medical education literature has broached the concept of hidden curriculum as a phenomenon that could have a negative influence on student learning [21]. However, even though it may be unintended, the unplanned curriculum may have positive benefits or experiences for the learner. Incidental experiences can sometimes lead to what Mezirow has termed "transformative learning," in which a disorienting dilemma brought about by an experience causes us to reflect on our own personal meanings and understandings, which can lead to more reflective and deeper learning [22-23]. The nature of the experiences and perspectives arising from the COVID-19 pandemic, and the effect on the learnings of postgraduate trainees, have not been explored in detail. Kumar et al. [24] suggest it is important to reflect on the value of the educational experiences that occurred during the pandemic so that helpful aspects can be retained or integrated into training programs in the future. The purpose of this phenomenological study was to explore postgraduate trainees' experiences with coping, managing, and learning during the COVID-19 pandemic.

## Materials and methods

Phenomenology is a qualitative research methodology, which emphasizes participants’ perceptions, feelings, and experiences as the paramount object of the study. The goal is to search for the ‘essential structures’ of the phenomenon by interviewing, in-depth, many individuals who have experienced the phenomenon [25]. This study adopted a phenomenological approach to explore postgraduate trainees’ experiences in coping, managing, and learning during the COVID-19 pandemic. Phenomenological research seeks to answer the question, "What is the essence of the experience of this phenomenon for those who experience it?" [26]. This study used a descriptive phenomenological approach, focusing less on the interpretations of the researcher and more on a description of the experiences of participants [27]. The use of a descriptive phenomenological approach allows researchers to explore, analyze, and describe a phenomenon while maintaining its richness, breadth, and depth [28]. Edmund Husserl, the founder of phenomenology, introduced the idea of descriptive phenomenology in part to counter the positivist paradigm, a deductive approach to exploring social reality [26]. Husserl argued that no assumptions or philosophical or scientific theory should inform a descriptive phenomenological inquiry; instead, the focus should be on understanding a phenomenon as perceived by the individual’s consciousness [29].

Semistructured interviews of approximately 30 to 45 minutes were conducted with a stratified purposeful sample of postgraduate trainees across a range of postgraduate years of training (PGY) and working across different programs and health regions in Newfoundland and Labrador (NL), Canada. The research team constructed an original interview script (Appendix) for the study. The interview questions were open-ended and focused on postgraduate trainees’ well-being and coping supports during the COVID-19 pandemic, as well as their learning and training experiences. The recruitment of study participants and the interviews took place between May and October 2022. The letter of invitation to participate in an interview and the promotional poster were distributed via email to residents. The interviews were conducted via videoconferencing and were recorded, transcribed, and imported into NVivo 20 analytical software.

Data were analyzed using a thematic analysis technique based on descriptive phenomenology. The goal of this thematic analysis was to achieve an understanding of patterns of meanings from data on experiences [30]. Drawing on the process of thematic analysis that Sundler et al. [30] suggested for descriptive phenomenology, our data analysis began with getting familiar with the raw data with open-minded reading. Then, we searched for meanings of experiences, compared differences and similarities between meanings, and grouped meanings into themes. To enhance the trustworthiness of our study, multiple coders were involved in open inductive coding. First, two common transcripts were independently reviewed by three research team members, who then met to discuss patterns of meanings from the data and generated an initial coding schema. Then, a second-level coding was done using this initial coding schema by two other researchers who independently coded the same two transcripts for verification of the codes. Through this second round of coding, the team refined, expanded, and elaborated to produce a focused coding schema. Application of this coding schema was then applied to the remaining six transcripts through multiple rounds of coding that varied between deductive (applying the schema developed through inductive coding) to inductive (expanding, refining, and elaborating schema). Two members of the team collaboratively collated the coded transcripts, refined the coding schema, and identified common themes. 

The Newfoundland and Labrador Health Research Ethics Board (HREB) provided approval for this study, Reference # 2022.049. Participants provided informed written consent before participating in the interview and verbally provided consent again before the commencement of the interview.

## Results

Videoconferencing was used to conduct semistructured interviews with eight residents (*n* = 8), representing a variety of programs and postgraduate training years (Table 1). Several key themes emerged from the interview analyses, including trainee experience of the COVID-19 pandemic, adjustment and adaptation to changes, and positive impacts on postgraduate trainees’ future learning and careers.

**Table 1:** Respondents' demographic characteristics.

Respondents’ characteristics	n	%
Gender		
Male	4	50.0
Female	4	50.0
Postgraduate year		
PGY1	2	25.0
PGY2	1	12.5
PGY3	3	37.5
PGY4	1	12.5
PGY5	1	12.5
Program		
Diagnostic radiology	1	12.5
Family medicine	1	12.5
General surgery	1	12.5
Neurology	1	12.5
Pediatrics	1	12.5
Psychiatry	3	37.5

Postgraduate trainee experience of the COVID-19 pandemic

When asked to describe their experiences of working and training during the COVID-19 pandemic, postgraduate trainees described changes in the nature, volume, and scope of work; changes in work-life balance; limited learning experiences; and a lack of group cohesion. Postgraduate trainees discussed how the *normal* hardship of residency was intensified by public health measures, such as travel restrictions, isolation requirements, and mask mandates, which were implemented to stem the spread of COVID-19. Several recommendations were proposed that could prove beneficial for postgraduate trainees in supporting their mental health and well-being during the pandemic. These include creating and maintaining personal and professional relationships, balancing support within and beyond residency training and work; seeking counseling, mentorship, and debriefing opportunities; implementing mandatory wellness check-ins; and providing more flexibility and access to leave options and time off. Table 2 summarizes specific quotations from respondents, which reflect the various themes and subthemes that emerged.

**Table 2:** Resident quotes.

Themes: Subthemes	Resident quotes
Theme 1: Trainee Experiences	I think the hardest part as a medical trainee is identifying when it is time to reach for help. I do not know how you would implement it really, but some sort of a mandatory check- in. There are resources that are available as rescue resources, but I feel like a lot of the time by the time those are being utilized it is already kind of past the point … we have our Office of Learner Well-Being and Success, but you have to reach out to them … right after this I'm going to my program director meeting that I do once a year [to look] at all my evaluations and everything that's going on … That’s twice a year, but we could easily have an equivalent, mental health and wellbeing check in. It could be the same thing. Five minutes … there's maybe five screening questions and then it makes the resident for the first time look at themselves and say, oh, maybe I'm not doing so hot right now. That would be helpful. I do not think it would be difficult to roll out. [Resident #3] A day off as a day for your mental health and wellness. Having a couple of more flex days in the year would be very helpful … Especially for people who were from away; they could not go home and see family [there] needs to be some loose mechanisms put in place [where] they are not getting any academic penalties, [and can] take some time away, [and] ways to make it up through another means. [Resident #4]
Changes in the Nature, Volume, and Scope of Work	Our discipline turned into an emergency basis only … we ended up seeing cases that are a lot more complex and stuff, because people were presenting later with more serious issues … we ended up seeing even advanced cancers that we haven't even really seen in years. [Resident #6] The types of patients that you are treating were just the sickest of the sick because people were avoiding coming to the hospital and waiting until it was too late … whenever we would have a healthcare shut down … within a month you would get this huge influx. You just get slammed with all these super, super sick patients. [Resident #3] Because of the backup in the healthcare system, the lack of primary care, we are all feeling that. My work and my on-call duties have increased exponentially … The volume of patients that we see, especially on call, it has increased threefold … clinics, they are much harder to schedule, the volume is higher, but they are much harder to schedule and then you have issues with the technology and everything and then you are so busy. You have all these conflicting things coming at you, not that they were not there before, but that they seemed worse now. [Resident #2]
Changes in Work-Life Balance Positive Change	Most of us felt as though we had never really had any time to explore interests or do anything that was not academic … I actually picked up a bunch of new practices and wellness things throughout that break and many of my friends did as well. [Resident #2] The extracurricular stuff … like a master's program. That is something I did a couple of years ago during the first wave when I just had all this free time, because I was not at work as much … I never would have signed up for that had I not had that period of time to just take some time and think about my career goals. [Resident #3]
Limited Learning Experiences Lack of Networking and Informal Learning The Need to Acquire Specific Competencies	A lot of our learning, at least in my specialty, really comes from sitting next to someone and reviewing scans in live time … We did not really get that … usually, when they review a case, they will say, oh, this is common exam knowledge or this is something that comes up on the exam all the time … We could not even do our regular rotations … everything was shut down and just emergency only. That went on for a very long time … so we really lost our normal curriculum … We did not watch seniors. We did not have any senior residents. We were really our own island … it takes a village … even the technologist teaches a lot and other residents from other disciplines when we are on call and we are talking to them. But all of a sudden going to this no contact or limited contact … unfortunately, sometimes learning goes to the back when it comes to a public health emergency. [Resident #6] I was not able to go to any conferences in the first year. That was an entire year of not networking. People were able to do virtual electives and things like that, but in terms of actually being able to travel the opportunities were fairly slim and same with being able to do elective rotations. [Resident #5] No networking. You're basically unable to communicate effectively with your colleagues on a day-to-day basis, socially. I mean, everyone could basically still talk about the task at hand, but there's all this subtle [stuff] that you have in between patient interactions and outside of work that add to your development as a physician. And really add to, I don't know, your growth as a person or whatever. [Resident #1] …all of a sudden, we were not hitting benchmarks …I went and spoke to the program director and was told this is a phenomenon [within my discipline]. For your particular year, all across Canada, you are all on average 6 months behind where other cohorts were. [Resident #6]
Lack of Group Cohesion	Since we were new residents, we felt very disconnected from our resident group … How you would have gotten that support from your group would be through your [in-person] academic events schedule. Throughout the week you would meet up and you would get to have a chat after they are finished, or for social events, and we have neither … we didn't even have that informal 30 minutes together a week to chat, talk about an upcoming exam, or talk about how things are. [Resident #2] One of the main things [that] affected our group, is group cohesiveness … before we'd all work in the reading room together and stuff. When we went to these solo, just only coming in to do call, and trying to social distance from each other … you end up really almost losing sight of the other people who are working alongside you. This is a real negative thing. [Resident #6]
Theme 2: Trainee Adjustment and Adaption to Changes	The pandemic has been challenging for everybody, but I will say that leaders in the program have appreciated that it has been challenging and they have made it very clear that should anybody need any additional support or resources that would be possible. So, there has been kind of like an overarching message of support which I think is really important. [Resident #5] I had a lot more formal debriefs with staff than I ever had before the pandemic. Over a night on call that might have been considered … but nowadays we are a lot more like, let’s debrief. How do you feel about ___? And talking about it to bring other scenarios in. [Resident #3]
Theme 3: Positive Impacts on Postgraduate Trainees’ Future Learning and Career Emotional Intelligence Empathy and Compassion	I have had to care for a lot of people who are COVID-positive and you'd have to fully gown up here. Patients are coming from different backgrounds, you still have to maintain a certain amount of distance with the gown and stuff if they are not going to mask up or if they are not able to mask up. I think learning how to still be compassionate without relying on a lot of the physical touch and cues and proximity. I think that has been a positive. [Resident #2] I think it was obvious as a provider in the pandemic when we are in this together I felt like I had the same fear as the patients. So I felt like I was on the ground with them and I could understand where they were coming from … It helps me kind of relate to patients on that level and hopefully my future practice I'll be able to apply that. I think it gives me a bit more of an understanding of patient behavior and patients’ decisions in ways that I can help patients in the future. [Resident #3]
Improved Culture of Trainee Health and Safety	… you just do not call in sick unless you are dying. Now with public health measures, if you have a cough you do not come in to work. We have been forced to learn how to deal with people taking sick days and changing our culture around it. Not “guilting” people around it. [Resident #3] One thing that I have taken back from working during the pandemic is just being aware of looking after myself … I think it has really encouraged us to just take a step back and say, could this be COVID? Or could this be X, Y, and Z? What do I have to wear in order to protect myself? [Resident #5]
More Experience, Exposure, and Skill Ownership/Leadership	I would say the pandemic has really forced us to take a lot more ownership into our own education. For example, when academic sessions shut down, it was one of our residents that kind of got things going, bought our own zoom accounts, said let's go, we're going to do this, things became like that too. So residents claimed a lot of the roles that we didn't have in the past. We have had to take a lot more agency and leadership within ourselves, which has been an important thing. [Resident #3]
Importance of Virtual Technology	Our learning recovered completely and actually surpassed from anything it ever was … Since the pandemic hit and we got virtual learning on the go, we have been doing [regular journal clubs] every single month [and] have actually continued that to this day … [and with] a virtual component now, way more people are tuning in every day. We’re getting much more engagement from staff positions and everything because it is just so much easier to tune in for journal club at 8 PM. If you have small kids, you can still tune into journal clubs. [Resident #3]

Changes in the nature, volume, and scope of work

Significant changes in the nature, volume, and scope of resident work were reported. Not only was there a significant shift from in-person to virtual care, but disciplines also shifted to emergency coverage, patients were presenting with more severe conditions, and patient volumes were higher (Table 2).

Changes in work-life balance

Residents also reported changes in their experience of balancing work and life, as well as study and work. Some of these changes were positive, including the ability to multitask, advance career goals, pursue non-academic interests, and have more time for family. However, with these positives also came the negative. One trainee captured the double-edged consequence of these changes when describing the feeling of needing to always be available or being unable to *leave work* (Table 2):

*All switched over to the virtual format. On one hand, that was a good thing in the sense that it allows you to multi-task. I could clean the floor while I am listening to the lecture … [but] everyone is burnt out by it. Even though it is easier to have virtual meetings and academic half days, we just had too many meetings … There is more emphasis on you to always be available and so when you go home, you want to be able to turn off, but that is the time when most people can make meetings. Then you have evening meetings and weekend meetings. It is a big ask.* [Resident #4]

Another resident detailed the prevalence of residents encountering challenges with childcare:

*A lot of our residents have multiple children. Trying to be home, then all of a sudden the childcare, and just trying to keep up on that. It almost felt like we were not in residency for about 2 months.* [Resident #6]

Limited learning experiences

Residents recounted their experience of missing out on vital learning opportunities during the pandemic, including live or direct teaching and learning, training rotations, and opportunities to learn from other residents and staff. A lack of networking and informal learning from colleagues was also noted, both of which are highly valued for personal and professional development. Particularly within the context of postgraduate medical education, which takes a competency-based approach, residents were keenly aware of the need to acquire specific competencies to advance in their training and subsequent careers. Such limited learning opportunities and clinical exposure may negatively impact their ability to reach typical milestones and benchmarks within their training (Table 2).

Lack of group cohesion

Due to the shift from in-person to virtual education and training and the public health measures implemented in response to the pandemic, residents reported a lack of group cohesion and few opportunities to engage in team-building activities (Table 2):

*I do not think I have had that same, you know, camaraderie, workplace environment that people would normally have had when there's no masks, no mandates, no restrictions … [There has been] no real team building and being able to bond with your colleagues. I mean, there is that, but not as much as it would have been.* [Resident #1]

Postgraduate trainee adjustment and adaptation to changes

Residents described the nature and types of support they relied upon during the pandemic, and these often included family, friends, and peers. Due to public health measures and restrictions, these connections were often enabled by virtual and digital technologies, such as Facebook, text messages, FaceTime, and video or telephone calls. Residents also reported seeking support from their family physician, availing of leave or sick days, debriefing with staff, and engaging in a variety of activities as a means to support their mental health and well-being. These activities included spending time outdoors, walking, hiking, or connecting with nature; exercising regularly; saying *no* more often; taking courses related to physician leadership and self-reflection; and engaging in meditation and mindfulness. Several residents indicated that their Program Directors or leaders were also available for support if they were ever in need (Table 2).

Positive impacts on postgraduate trainees’ future learning and career

**Figure 1:**
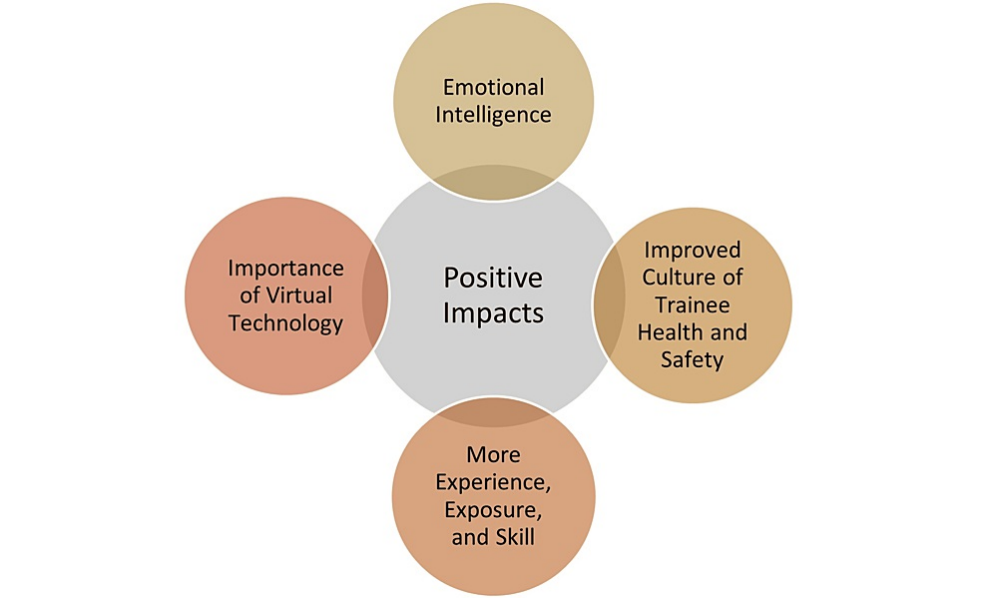
Positive impacts. Image credit: Vernon R. Curran.

A variety of positive insights and new learnings were reported by postgraduate trainees when describing their work and training experiences during the COVID-19 pandemic. Several subthemes emerged from the interviews regarding perceived positive or beneficial impacts on their learning and future careers, including emotional intelligence; improved culture of trainee health and safety; more experience, exposure, and skill; and the importance of virtual technology (Figure 1).

Emotional intelligence

Emotional intelligence includes the ability to understand and manage one’s own emotions, as well as recognize and influence the emotions of others [31]. Trainees described an increase in several key traits associated with emotional intelligence, such as self-awareness, self-regulation, empathy, and resilience:

*I really feel like I got to know myself a lot better. I know my response to stress is better and I know what to do to cope and recharge. That has probably been the biggest upside of the pandemic now. *[Resident #2]

Some trainees described feeling like they were more adaptable and resilient because of working and training during a pandemic:

*I think we have just adjusted without even noticing we were doing anything … I had a little bit more resilience and I came from a better place in terms of coping skills. *[Resident #2]

Some also discussed developing a greater sense of empathy and compassion (Table 2).

Improved culture of postgraduate medical trainees' health and safety

A slight culture change was also noted by trainees, in that their pandemic experience led to more acceptance of others taking time off and the importance of personal health and safety (Table 2).

More experience, exposure, and skill

Trainees described improved observation and nonverbal communication skills, more experience, and increased exposure to complex cases. For example:

*In some ways, I feel stronger clinically because of it. Because during the pandemic people were afraid of coming back to hospital. We were seeing the most complex cases on call and the highest volumes we ever saw. So, I feel like in terms of being able to manage the clinical day-to-day cases, the bread and butter, and even the more complex stuff, we got great exposure to that.* [Resident #6]

One resident even described how the rapid changes to their education and training sparked a sense of ownership and leadership in their education (Table 2).

Importance of virtual technology

Several trainees described virtual learning as something that improved learning, increased engagement and attendance, and enabled regular meetings and academic half-days. Several trainees also noted an increased level of comfort and experience with virtual care and the importance of virtual care delivery within NL healthcare (Table 2):

*I think there is certainly a role for virtual care, and I do not think I was overly comfortable doing that. I had never done it really … So, I've definitely gained a better comfort with that. *[Resident #2]

## Discussion

Protecting HCWs emerged as an important component of public health measures for addressing the COVID-19 pandemic. Numerous authors have recommended that special efforts to promote the psychological well-being of postgraduate trainees during COVID-19 be enhanced with psychosocial support systems. These systems should acknowledge the additional stress related to COVID-19 within the context of already challenging postgraduate training [2,15]. The findings from our interviews indicate that trainees reported seeking support from their family physician, availing of leave or sick days, debriefing with staff, and engaging in a variety of self-care and self-learning activities to support their mental health and well-being during the pandemic. Despite this, trainees also described feeling isolated from and unable to access usual support, such as family and friends who were often not located within their region or province of study. This highlights the importance of exploring and maintaining awareness of the background of trainees - particularly of what support or support network they have and how accessible these are to them.

Interview findings also suggest that additional support may need to be provided to those without access to appropriate and preferred sources of support during a healthcare crisis, such as the COVID-19 pandemic. Since postgraduate trainees experienced a lack of group cohesion and opportunities to interact and network with staff, peers, and colleagues, it will be important to focus on team building. This focus aims to enhance camaraderie within and between disciplines and professions, especially as public health measures are lifted and work and social routines return to normal. Additional suggestions included administrative recommendations for program and systemic change, such as providing protected time for socials and events, implementing mandatory wellness check-ins, and allowing more flexibility and access to leave options and time off. One study participant described a perceived inability to disconnect from work, as residents were expected to either be on standby or engage in activities outside of regular hours, including answering work calls, emails, or other work-related communications. Another resident also discussed a perceived increase in the amount and frequency of meetings. Meetings serve different purposes, ranging from informal social gatherings to formal program sessions. While there was a slight decrease in the average length of meetings during the pandemic, the average number of meetings rose significantly compared to the pre-COVID-19 period. Ineffective meetings that consume time or are perceived as a suboptimal use of time can negatively impact psychological, physical, and mental well-being.

Zaçe et al.’s [10] systematic review revealed that interventions addressing mental health issues in HCWs during pandemics could be grouped into four categories: (1) informational support (training, guidelines, and prevention programs); (2) instrumental support (PPE and protection protocols); (3) organizational support (manpower allocation, working hours, reorganization of facilities/structures, and provision of rest areas); and (4) emotional and psychological support (psychoeducation and training, mental health support team, peer support and counseling, therapy, digital platforms, and tele-support). Other recent literature focused on the provision of informational support and emotional and psychological support to individual HCWs. As examples, these supports could encompass the promotion of psychological first-aid programs, digital self-care packages, peer support, telehealth counseling, and other such strategies that promote healthy coping strategies, resilience, personal agency, wellness, and self-care [1-2,11-12,14,32-38]. Medical educational program design may also be enhanced to promote both learning and medical learners' well-being. Such enhancements may relate to teaching and assessment, the learning environment, and faculty and staff development. Examples might include designing curricula that promote peer support and progressive levels of challenge to learners; designing assessment tasks to foster well-being and learning; providing physical health promotion initiatives; and training faculty and staff on medical learners' well-being and how to address well-being concerns.

Due to the shift from in-person to virtual learning and the public health measures implemented in response to the pandemic, not only did trainees report a lack of group cohesion and opportunities to engage in team-building activities, but they also missed live or direct teaching and learning opportunities, training rotations, and opportunities to learn from other trainees and staff. Trainees were aware of the need to acquire specific competencies to advance in their training and subsequent careers and were concerned about how such limited learning opportunities and clinical exposure would impact their ability to reach typical milestones and benchmarks. As the impacts of the pandemic emerge over time, this may represent a larger problem within the context of competency-based postgraduate medical education.

Kumar et al. [24] indicated that the pandemic had a significant global impact on postgraduate medical education with many switching to virtual learning and online webinars to enable continued teaching and learning. Chasset et al.’s [39] review found that online learning was the most frequently used method to ensure continued postgraduate medical education during COVID-19, with a general positive level of trainee satisfaction with this approach. The rapid and unprecedented changes to their education and training also led to several positive changes in trainees' educational and personal development. An increase in self-directed learning skills was noted, with trainees reporting a greater sense of ownership and leadership of their education. Virtual learning also reportedly improved their learning, increased engagement and attendance, and enabled regular meetings and academic half days when usual in-person learning was not available.

Postgraduate trainees reported increased confidence and experience with virtual care, as they realized the importance of virtual care delivery within the NL healthcare system. Kumar et al. [24] also noted the benefit of exposure to virtual care delivery and the learning of specific communication skills and diagnostic acumen specific to this delivery modality for trainees during the pandemic. A shift in the culture surrounding trainees' health and safety was also reported, as trainees noted a move away from traditional work-life culture to more acceptance of others taking time off and the importance of personal health and safety. Additionally, trainees demonstrated increased emotional intelligence (e.g., self-awareness, empathy, and compassion), which may enhance learning and the delivery of patient-centered care.

A limitation of this study may be the smaller number of respondents interviewed; however, the respondent sample was reflective of gender diversity and respondents did reflect a variety of postgraduate program areas and year of study. Furthermore, there was a level of saturation found around particular subthemes during the interview analyses phase of the study.

## Conclusions

The learning experiences of postgraduate trainees during the pandemic were altered abruptly with a shift to virtual learning, with some impact on normal clinical training exposure. Residents felt isolated from their peers and normal training routines. However, several areas of learning opportunity arose because of the pandemic. Residents gained greater experience with digital technologies by learning online and delivering virtual care, which enabled the development of self-directed learning and virtual care delivery ability. Virtual technologies continue to be used post-pandemic in many program areas. Thus, virtual care education is a key area requiring further attention in postgraduate training programs, and it will be important to recognize the benefits of these new ways of learning to enhance the ongoing and future experiences of postgraduate trainees.

The determinants of well-being are multifactorial, including aspects of the individual’s physical and mental health, sense of fulfillment from work, social inclusivity and quality of the learning and working environment, larger community, and cultural norms. Learning organizations often target various determinants under their control, such as interventions focused on individuals identified as having lower well-being, or preventative interventions aimed at maintaining the well-being of the wider learner population. Unfortunately, to date, interventions to improve medical learners' well-being have been poorly studied, with generally variable quality evidence for the effectiveness of interventions that improve the well-being of learners, faculty, and staff at both the individual and the organization/systems levels. Future work, including ongoing work around the adoption of the Okanagan Charter across Canadian medical schools, holds much potential to foster healthier learning and working environments for all learners.

## Appendices

Appendix

**Table 3:** Interview script.

Can you describe how your experiences as a *trainee* have been affected as a result of the COVID-19 pandemic?
Prompts:
How have your specific *educational* and/or *training* experiences as a learner been affected?
How have specific *clinical* responsibilities you were assigned been affected?
What have been some specific challenges you have encountered?
With work-life balance? Your emotional and/or mental health? Your physical well-being? With your personal and/or social life?
Have you *learned* anything new or different on a professional and/or personal level as a result of training and working during a public health emergency that has helped you develop as a healthcare provider?
Prompts:
Have you gained any useful *insights* from your educational, professional, and/or personal experiences during COVID-19?
How do you feel your experiences as a trainee during COVID-19 will affect you as a healthcare provider in the future?
Prompts:
What have been some of the *positive* aspects of your experiences during COVID-19 that will help you in your future professional career?
What have been some of the *negative* aspects of your experiences during COVID-19 that could hinder you in your future professional career?
How would you describe your experiences in adjusting and adapting to the changes in your training and work responsibilities during COVID-19?
Prompts:
Do you feel you were well prepared to manage and lead these changes?
Were there any insights, knowledge, skills, or abilities that might have enhanced your adaptability and agility to cope or even thrive in response to stress?
Based on your personal experience what would be your advice to another postgraduate trainee about the value of immediate support such as family and friends during COVID?
What have been sources of strength or support for you during COVID-19?
Prompts:
How have you sought support for any stress, anxiety, or other negative emotions you may have experienced as a result of the changes brought on by COVID-19?
What types of support systems or programs have been particularly helpful to you?
If you were to build a program of support systems for future trainees experiencing a public health emergency, what do you feel would be most beneficial and useful?
